# Tuning the Quantum Properties of ZnO Devices by Modulating Bulk Length and Doping

**DOI:** 10.3390/e24121750

**Published:** 2022-11-29

**Authors:** Zheng Fan, Gui-Qin Li, Gui-Lu Long

**Affiliations:** 1 State Key Laboratory of Low-Dimensional Quantum Physics and Department of Physics, Tsinghua University, Beijing 100084, China; 2 Frontier Science Center for Quantum Information, Beijing 100084, China; 3 Beijing Academy of Quantum Information Sciences, Beijing 100193, China

**Keywords:** quantum calculation, molecular device, density of state, electronic transmission

## Abstract

The quantum transport properties of ZnO devices with five different bulk configurations are investigated with numerical methods. The calculation results reveal that the transport property at a higher energy range can be tuned by changing the length of central scattering. By substituting some Zn atoms with Cu atoms, it is found that the doped Cu atoms have an obvious effect on the quantum properties at the entire energy range investigated, and could result in different transmission. The properties of ZnO devices are also influenced by the doping positions of Cu atoms. The tuning mechanism relies on the shifting of carrier distributions in the scattering center of the device.

## 1. Introduction

Molecular-level quantum devices have great potential in the future. The investigation and fabrication of molecular-level devices have been performed for decades [1,2,3,4,5,6,7,8]. An interesting type of molecular level quantum devices consists of two terminals and the extended scattering in the middle [9,10,11,12,13,14,15,16]. Since more and more new materials can be synthesized with the development of material technology, the design of new molecular-level devices is under rapid progress by finding new materials as the central scattering of the device. The tuning technique has become a powerful tool in material and device design. By controlling film thickness and electron doping, the superconductivity in alkali fulleride films can be well tuned [17]. Atomic layer superconductivity can be tuned by Rashba/Zeeman-type coupling [18]. Molecular-level devices were usually considered to function as below 1 nm classical computation units and they also have potential applications in quantum information science. Molecular-level devices are possible substitutes for qubits [19,20], which are the basic units of a quantum computer. The intermediate controller switching the interaction between qubits and environment can also be made by molecular devices due to their electrical properties [21]. Moreover, molecular-level devices can assist in realizing non-demolition measurements [22]. Tuning the quantum properties of molecular-level quantum devices is vital to materializing these applications.

Our study focuses on ZnO-based molecular-level devices. It is well known that ZnO crystal has several typical structures, such as NaCl structure, zinc-blende structure, etc. [23,24]. Furthermore, ZnO also shows advantages over other oxides in many physical characteristics, such as stability, optoelectronic properties and etc. [24,25]. It has been demonstrated experimentally that the electronic properties of ZnO can be obviously tuned by doping Cu atoms [26], increasing the adjustability of ZnO-based devices. Thus, they have plenty of applications, including optoelectronic devices, field effect transistors and etc. [27,28]. Among the most exciting progress is molecular-level quantum devices using ZnO as an electron-transporting layer for its excellent electron mobility [29]. Quantum dot systems containing a ZnO layer can both serve as light-emitting diodes [30] and photodetectors [31]. Various fabrication techniques [32,33] have been developed for ZnO-based molecular devices toward the applications to quantum information processing. In this paper, we investigated the quantum properties of one layer ZnO devices and found its electronic properties can be changed by modulating the length of the central scattering and doping Cu atoms.

**Figure 1:**
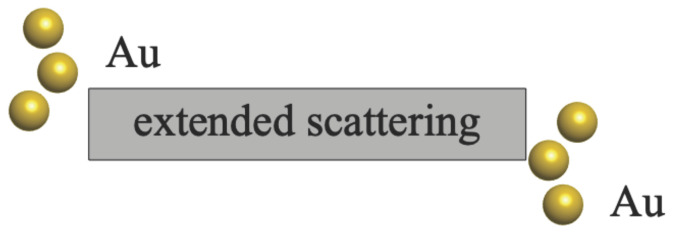
A ZnO device consists of two parts, an extended scattering and the terminals. The extended scattering is made up of the ZnO central scattering and bridge S atoms. Terminals of the device are gold electrodes made by Au (111) atoms. Only the first layer of each electrode with three Au atoms is drawn.

To study the electronic property and tuning effect of the ZnO device, we focused on the NaCl structure, and consider single layer crystal. Gold electrodes (Au (111) direction) were chosen as two terminals, as depicted in Figure 1.

Every terminal consists of three layers, and three, seven, and three Au atoms at each layer respectively. Au (111) films are the commonly used electrodes in molecular level devices [34], and such choice is also consistent with experiments. Other orientations of Au will result in different Fermi energy. The ZnO layer is connected with the first layer of Au atoms. The chemical bond between S and Au atoms ensures stable contact with the electrodes. The electronic properties and tuning effects are characterized by the density of state (DOS) and transmission properties of devices. Our study was carried out by using Green’s function and tight-binding methods [24,34,35].

## 2. Materials and Methods

**Figure 2:**
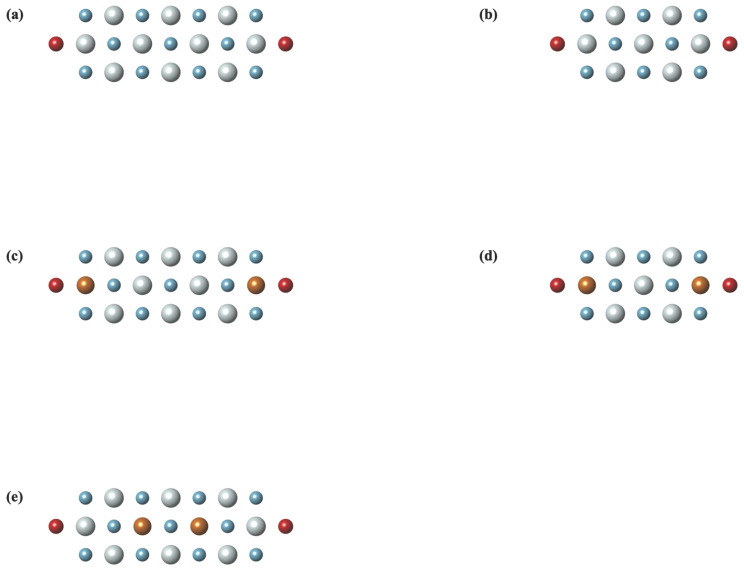
Extended scattering configurations of different ZnO quantum devices. (**a**,**b**) show undoped ZnO central scattering with different lengths. The red ball represents the bridge atom S at left and right end contacting with the gold electrodes. In the central scattering material, blue ball is the O atom, while light grey ball is the Zn atom, and in the doped cases (**c**–**e**), the Cu atom is represented by the bronze ball.

To study the tuning effect of size and doping on the transmission coefficient (TE) of ZnO quantum devices, ZnO five different configurations of extended scattering were considered sequentially, as shown in Figure 2. The geometry parameters of ZnO devices were set as: the bond length of Zn-O was 1.895 Angstrom, and Au-Au was 2.885 Angstrom in Au electrodes; the distances between the bridge S atom and the two side atoms Zn and Au were 1.97, 2.53 Angstrom, respectively. Figure 2a corresponds to 21 atoms in the ZnO layer, and Figure 2b corresponds to 15 atoms. The length of the central scattering along the direction of electrodes is approximately 0.7 to 1.2 nm. The bulk size of the devices is characterized by the number of atoms in their central scattering. Figure 2c has the same geometry parameters as Figure 2a, except that two Zn atoms bounded with S atoms are substituted by two Cu atoms. Similar relations lies between Figure 2a,b. Figure 2e shows the same geometry parameters as Figure 2c, except that the positions of the doped Cu atoms are shifted to the middle of the central scattering. The geometry of these five quantum devices is then optimized using G09, DFT-B3LYP/6-311G basis.

The tight-binding method is widely used in the study of electronic properties of molecular level devices [36,37]. This method shows advantages in direct physical insight with economic computation routine [38]. However, due to its approximation nature, the tight-binding method lacks accuracy in case of charge transfer, relaxation of coordinates and overlap between distant atomic orbitals. Compared with other methods that are more accurate, the tight-binding method is still proven successful in the prediction of electronic properties of molecular systems [38,39,40]. In our study, the geometry parameters of the ZnO devices are optimized to improve the accuracy of the numerical results of tight-binding methods. By the Green’s function and tight-binding method [24,34,35], the transmission coefficient can be obtained from the retarded Green’s function matrix *G*, as

(1) $$T=Tr(\Gamma_{1}G\Gamma_{2}G^{†}),$$

where the trace is over all the orbitals of the central scattering, and $\Gamma_{1}$, and $\Gamma_{2}$ are the imaginary part of the self-energy of two electrodes, respectively, which describe the coupling between the bulk and the electrodes due to the energy level broadening. For the five different ZnO quantum devices with the optimized structures, we numerically calculate their density of states and the electronic transmission [34].

The numerical calculation is carried out by running the Huckel-IV program [35], where the structure parameters of a ZnO quantum device are input to obtain the Huckel matrix and the overlap matrix of the neutral molecule. These results are then used to calculate the overlap matrix and coupling matrix between the device and the gold electrodes giving the coupling factor equal to 1. Finally, with the extended Hückel theory, these matrices together with the surface Green’s function obtained for the tight-binding Hamiltonian are sufficient to compute the density of state and electronic transmission of the molecular device. The total energy range is from −14 to −6 eV.

## 3. Results

### 3.1. The DOS of Au-S-ZnO-S-Au Device

**Figure 3:**
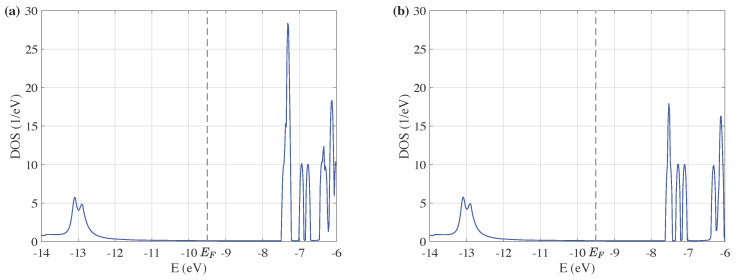
Density of state of ZnO quantum devices with different size as the energy ranges from −14 to −6 eV: (**a**) DOS of ZnO with 21 atoms, and (**b**) DOS of ZnO with 15 atoms.

Figure 3a,b give the density of state of the two ZnO devices corresponding to structures of Figure 2a,b, respectively. The Fermi energy $E_{F}$ of Au (111) in the simulation is selected to be −9.5 eV [34,35], since Hückel energy levels are 4 to 5 eV lower than the work function of gold. The same value of $E_{F}$ is also taken in later results.

The DOS in both cases is small when energy is from −10.5 eV to −7.5 eV. As the size of the central scattering decreases, the DOS distribution is almost the same, except that some peaks of the DOS above −7.5 eV decrease as the size decreases. The gap between peaks on either side of the Fermi energy is almost equal in these two cases. Therefore, the length of the central scattering has little influence on the electron transport properties near the Fermi energy.

### 3.2. The DOS of Au-S-ZnO-S-Au Devices with Doped Cu

**Figure 4:**
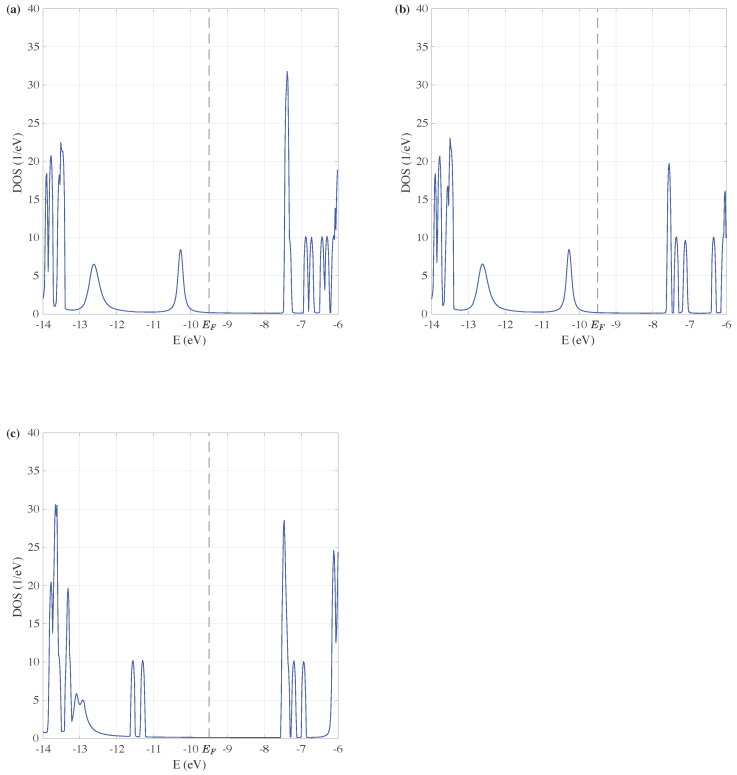
Density of state of ZnO quantum devices with doped Cu atoms as the energy ranges from −14 to −6 eV. In (**a**), two Cu atoms substitute two Zn atoms which connect to the bridge S atom, and there are 21 atoms in the central scattering. In (**b**), the doping position is the same as (**a**), but the central scattering reduces to 15 atoms. (**c**) has the same size as (**a**), but the doping position is in the middle of the central scattering.

Three ZnO devices with doped Cu atoms are shown in Figure 2c–e, their geometry parameters of atom bonds are the same as the ZnO devices without doping, then optimized using G09, DFT-B3LYP/6-311G basis. The DOS of these devices with doped Cu are shown in Figure 4a–c.

The gap between peaks on either side of the Fermi energy is smaller in the doped devices compared with undoped cases in Figure 3, indicating that the DOS of ZnO devices with doped Cu atoms has more activity properties. This is the consequence of the increase of carrier concentration through doping. It is also worth noting that there are more oscillating peaks of the DOS within the lower energy region. In Figure 3a, the intensity of DOS is relatively small between −12.0 eV to −10.0 eV, but in Figure 4a, the intensity increases obviously, even a peak appeared near −10.2 eV. Thus, the doping of Cu atoms improves the electron transport properties of ZnO devices.

By comparing Figure 4a,b, it is found that the tuning effect of size on the DOS of ZnO devices with doped Cu atoms is similar to the undoped cases. The DOS of Cu-doped ZnO devices with different sizes varied only at higher energy levels. The tuning effect of the doping position on the DOS of ZnO devices is investigated by comparing Figure 4a,c. The peaks between −13 eV to −10 eV split to narrower peaks in Figure 4c, and they are compressed to lower energy region, leaving a larger gap near the Fermi energy. It can be seen that the position of the doped Cu atoms is also of significance in the tuning effect on electron transport properties of ZnO devices.

### 3.3. The Transmission Properties of ZnO Devices

**Figure 5:**
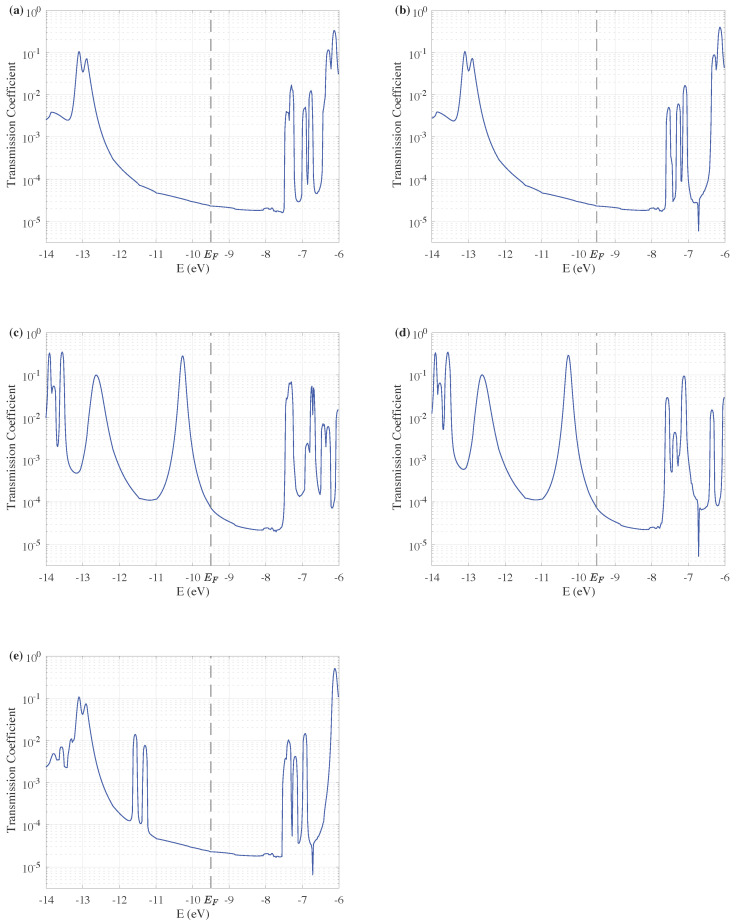
Transmission of ZnO quantum devices with different configurations as the energy ranges from −14 eV to −6 eV. The transmission is plotted on a logarithmic scale. (**a**–**e**) correspond to structures shown in Figure 2a–e, respectively.

Figure 5a–e show the TE of the five ZnO devices corresponding to configurations in Figure 2a–e, respectively. The carrier distribution in the central scattering plays a vital part in determining the TE of the ZnO devices. Comparing Figure 5a with Figure 5b and Figure 5c with Figure 5d, it is found that the size of the ZnO devices can affect on TE in higher energy levels. As the length of the central scattering decreases, the number of peaks above −7.5 eV is slightly reduced. Yet the change of carrier distribution due to doped Cu atoms can affect the entire energy range. It is shown through the comparison between Figure 5a,c that the increase in electrons in the central scattering obviously increases the transmission coefficient near the Fermi energy. The TE of the ZnO devices doped with Cu atoms at different positions is quite different. By comparing Figure 5c,e, when the positions of doped Cu atoms are shifted from the edge to the middle of the bulk, transmission near the Fermi energy reduces and the gap between peaks on either side of the Fermi energy is increased, though the transmission properties are still better than the undoped devices.

## 4. Discussion and Conclusions

We have numerically computed the DOS and TE with energy range from −14 eV to −6 eV of five different ZnO quantum devices by tight-binding methods. The electron transmission properties can be controlled by tuning the size of the ZnO central scattering or substituting some Zn atoms with Cu atoms. As the length of the central scattering decreases, DOS in the energy range −7.5 eV to −6 eV also decreases, and thus transmission in the higher energy region can be tuned by the size of the ZnO quantum device. The doping of Cu atoms affects the DOS and TE of the entire energy range. Firstly, the doped Cu atoms significantly increase the DOS in the energy range −14 eV to −13.5 eV, though slightly decrease the DOS in the higher energy range. Transmission near the Fermi energy is also improved by doping. Thus the transmission of the ZnO quantum device can be tuned by doping Cu atoms. Secondly, different positions of the doped Cu atoms will result in different transmission properties. The mechanism of these tuning effects is to change the distribution of carriers. The density of the state almost resembles the distribution of the transmission coefficient if it is noted that the latter is plotted on a logarithmic scale. However, the peaks do not match exactly the density of the state and the transmission coefficient due to the characteristics of the wave function, which are not explicitly considered in our study. In summary, the ZnO quantum device requiring different transmission and density of states distributions can be tuned by the length of bulk and doping of Cu atoms. Further study will focus on the dynamical properties of ZnO-based devices such as quantum conductance, quantum efficiency and quantum current, as well as the tuning effect of size and doping in other molecular quantum devices, and their application as the links in quantum computing devices and sensors.

## Data Availability

Not applicable.
